# Interactions between anaerobic ammonium- and methane-oxidizing microorganisms in a laboratory-scale sequencing batch reactor

**DOI:** 10.1007/s00253-019-09976-9

**Published:** 2019-06-21

**Authors:** Karin Stultiens, Simon Guerrero Cruz, Maartje A. H. J. van Kessel, Mike S. M. Jetten, Boran Kartal, Huub J. M. Op den Camp

**Affiliations:** 1 Department of Microbiology, IWWR, Radboud University, Heyendaalseweg 135, 6525 AJ Nijmegen, The Netherlands; 2 Soehngen Institute of Anaerobic Microbiology, Heyendaalseweg 135, 6525 AJ Nijmegen, The Netherlands; 3 Microbial Physiology Group, Max Planck Institute for Marine Microbiology, Celsiusstraße 1, 28359 Bremen, Germany

**Keywords:** Anaerobic methane oxidation, Anammox, Wastewater treatment, Methylomirabilis, Methanoperedens

## Abstract

The reject water of anaerobic digestors still contains high levels of methane and ammonium that need to be treated before these effluents can be discharged to surface waters. Simultaneous anaerobic methane and ammonium oxidation performed by nitrate/nitrite-dependent anaerobic methane-oxidizing(N-damo) microorganisms and anaerobic ammonium-oxidizing(anammox) bacteria is considered a potential solution to this challenge. Here, a stable coculture of N-damo archaea, N-damo bacteria, and anammox bacteria was obtained in a sequencing batch reactor fed with methane, ammonium, and nitrite. Nitrite and ammonium removal rates of up to 455 mg N-NO_2_^−^ L^−1^ day^−1^ and 228 mg N-NH_4_^+^ L^−1^ were reached. All nitrate produced by anammox bacteria (57 mg N-NO_3_^−^ L^−1^ day^−1^) was consumed, leading to a nitrogen removal efficiency of 97.5%. In the nitrite and ammonium limited state, N-damo and anammox bacteria each constituted about 30–40% of the culture and were separated as granules and flocs in later stages of the reactor operation. The N-damo archaea increased up to 20% and mainly resided in the granular biomass with their N-damo bacterial counterparts. About 70% of the nitrite in the reactor was removed via the anammox process, and batch assays confirmed that anammox activity in the reactor was close to its maximal potential activity. In contrast, activity of N-damo bacteria was much higher in batch, indicating that these bacteria were performing suboptimally in the sequencing batch reactor, and would probably be outcompeted by anammox bacteria if ammonium was supplied in excess. Together these results indicate that the combination of N-damo and anammox can be implemented for the removal of methane at the expense of nitrite and nitrate in future wastewater treatment systems.

## Introduction

Aerobic wastewater treatment systems are energy-intensive and costly, as aeration for the removal of organic carbon and oxidation of ammonium requires large amounts of oxygen (Jetten et al. 1997). Furthermore, for complete nitrogen removal by denitrification, the addition of an external electron donor is needed (Kartal et al. 2010). In this respect, anaerobic treatment systems offer many advantages such as biogas production in the form of methane, which can be used to generate electricity. In addition, as anaerobic microbes grow slower, the sludge production in these systems is lower compared with that in aerobic treatment systems. However, the reject water of anaerobic treatment systems is still rich in reduced compounds, such as methane and ammonium. While there are several established processes that can be used to remove ammonium from the effluents of these anaerobic systems, currently there are no established biological methods to remove dissolved methane, resulting in the release of this potent greenhouse gas into the atmosphere (van Kessel et al. 2018).

In the past few decades, anaerobic microbial processes consuming ammonium and methane that depend on nitrite and/or nitrate reduction have been discovered and described (Mulder et al. 1995; Haroon et al. 2013; Raghoebarsing et al. 2006). The anaerobic ammonium oxidation (anammox) process is performed by microorganisms that use nitrite as their terminal electron acceptor, which produce N_2_ and nitrate (Strous et al. 1999). Combined with partial nitrification, this process is already applied worldwide in wastewater treatment and has shown to be an efficient and cost-effective system for nitrogen removal (Kartal et al. 2010; Lackner et al. 2014). Nitrate/nitrite-dependent anaerobic methane oxidation (N-damo) by a consortium of bacteria belonging to the NC10 phylum and methanotrophic archaea related to the ANME-2d clade was first described in 2006 (Raghoebarsing et al. 2006). The N-damo bacteria, named “*Candidatus* Methylomirabilis oxyfera,” were shown to use nitrite as an electron acceptor for methane oxidation, while the N-damo archaea prefer nitrate (Ettwig et al. 2008; Haroon et al. 2013; Hu et al. 2011). These ANME-2d clade archaea, named “*Candidatus* Methanoperedens nitroreducens,” reduce nitrate to ammonium via nitrite and oxidize methane via reverse methanogenesis to carbon dioxide (Arshad et al. 2015; Ettwig et al. 2016; Haroon et al. 2013; Vaksmaa et al. 2017; Gambelli et al. 2018). The production of nitrite and ammonium makes these archaea suitable partners for both *Ca.* Methylomirabilis and anammox bacteria (Arshad et al. 2015; Haroon et al. 2013; Meng et al. 2016; Shen et al. 2017). Indeed, studies have shown that anammox and *Ca.*Methylomirabilis-like bacteria and *Ca.*Methanoperedens-like archaea can be cultured together in laboratory-scale membrane reactors, together removing methane, ammonium, nitrite, and nitrate from the influent (Ding et al. 2017; Lu et al. 2017; Shi et al. 2013; Xie et al. 2017a, b). In addition to cooperation, these organisms may also compete with each other under substrate limitation (Luesken et al. 2011a, 2011b; Arshad et al. 2017; Guerrero-Cruz et al. 2019; Welte et al. 2016). *Ca.* Methylomirabilis sp. and *Ca.* Methanoperedens sp. both use methane, while *Ca.* Methylomirabilis sp. and anammox bacteria are both dependent on nitrite. In previous studies of the N-damo process, methane was added via a membrane system. In most anaerobic wastewater treatment systems, methane will be present as dissolved gas in the bulk liquid. In addition, biofilm growth on the membranes needs to be carefully monitored and controlled, to optimize the N and C removal capacity of the system (Chen et al. 2015; Wu and Zhang 2017). This might pose a challenge to control such a system when applied for full-scale wastewater treatment, as influent concentrations of substrates and temperatures vary considerably over time. Therefore, other biomass retention systems might be more practical in full-scale use.

Here, we describe an anammox/N-damo coculture in a laboratory-scale sequencing batch reactor (SBR) system that we used to study the microbial interactions between the three involved functional clades of *Ca.* Methanoperedens, *Ca.* Methylomirabilis, and anammox bacteria. Specific consumption rates of ammonium, nitrite, nitrate, and methane were determined using stable ^15^N isotopes in reactor and ex situ batch assays to determine the contribution of the different microbial groups to substrate removal. Community composition was monitored with fluorescence in situ hybridization (FISH) with specific probes for the three different groups.

## Materials and methods

### Establishment of an anaerobic methane- and ammonium-oxidizing coculture

An anaerobic methane-oxidizing enrichment culture (1.6 L; Ettwig et al. 2008) was used to inoculate a 3-L sequencing batch reactor (SBR) equipped with pH and dissolved oxygen sensors and connected to an ADI1010 bio-controller (Applikon Biotechnology BV, Schiedam, The Netherlands). Every SBR cycle comprised 10 h and 40 min of constant medium supply, 20 min of settling, and 1 h of removal of excess liquid. During the filling period, the culture was kept anoxic by continuous flushing with CH_4_/CO_2_ (95%/5%, 10 mL min^−1^). The gas effluent was discharged via a 5-L bottle, which acted as a “gas buffer” to further minimize air incursion from the atmosphere. At the start, synthetic medium containing 0.5 mM NH_4_Cl, 1.5 mM NaNO_3_, and 10 mM NaNO_2_ was added continuously. The medium further contained 0.3 g KH_2_PO_4_, 0.2 g CaCl_2_·2H_2_O, 0.2 g MgSO_4_·7H_2_O, 0.14 mg ZnSO_4_·7H_2_O, 0.06 mg CoCl_2_·6H_2_O, 0.1 mg MnCl_2_·4H_2_O, 0.4 mg CuSO_4_, 0.09 mg NiCl_2_·6H_2_O, 0.007 mg H_3_BO_3_, 0.01 mg SeO_2_, 0.01 mg Na_2_WO_4_·2H_2_O, 0.05 mg Na_2_MoO_4_, 0.01 mg CeCl_2_, and 2.5 mg FeSO_4_·7H_2_O per liter. Trace elements were added using NTA-chelated stock solutions. The medium vessel was continuously flushed with Ar/CO_2_ (95%/5%, 10 mL min^−1^). The pH was maintained at 7.3 by KHCO_3_ addition. The influent flow rate was adjusted based on the nitrite removal capacity of the culture. The SBR was stirred at 80 rpm by means of a turbine stirrer, and the temperature was kept constant at 30 °C with a heating blanket. During the removal period, the liquid volume (1.6 L) in the bioreactor was maintained by a level-controlled effluent pump. After reaching stable performance, the culture volume was reduced to 1.1 L upon settling of the biomass. Subsequently, on day 111, 500 mL of *Kuenenia stuttgartiensis* enrichment culture was added (Kartal et al. 2010). Ammonium and nitrite in the medium were gradually increased (up to 20 mM NH_4_^+^ and 40 mM NO_2_^−^) following the increasing consumption of these substrates by the biomass of the culture. Influent and effluent samples were taken on a regular basis and centrifuged for 5 min at maximal speed, and the supernatant was stored at −20 °C until further analysis.

### Reactor assay—anaerobicmethane-oxidizing activity

On day 105, before the addition of *K. stuttgartiensis*, the nitrite and methane consumption rate of the anaerobic methane-oxidizing enrichment culture was determined. The SBR cycle, influent flow, and CH_4_ inflow were stopped. The reactor was flushed with Ar/CO_2_ until the CH_4_ concentration in the headspace was approximately 8% (*v*/*v*). Subsequently, the headspace was sealed and the reactor was supplemented with 0.4 mM NO_2_^−^. Methane and nitrite samples were taken every hour.

### Reactor assay—substrate turnover and dinitrogen and nitrous oxide gas production

Turnover rates of ammonium, nitrite, and nitrate in the anaerobic methane- and ammonium-oxidizing coculture were determined during two reactor assays on day 520 and day 524. After the removal phase of the SBR cycle, methane inflow was stopped and the headspace of the reactor was sealed. Inflow of medium containing 20 mM NH_4_^+^, 40 mM NO_2_^−^, and 1.5 mM NO_3_^−^ was continued during the assay. After 1 h, the medium was replaced with medium containing ^15^NO_2_^−^ (all substrate concentrations were as described above). Gas and liquid samples were taken every 30 min.

### Batch assay—potentialmethane- and nitrogen-removing activity

The headspace of 60-mL serum bottles containing 25 mL medium (see the above section) with substrates (Table 1) supplemented with 5 mM 3-morpholinopropanesulfonic acid (MOPS) buffer (pH 7.4) was exchanged with argon by 5 cycles of vacuum/gassing followed by 10 min flushing with argon. Subsequently, 5 mL biomass from the reactor was added. The headspace was evacuated and exchanged with argon twice to remove any remaining methane from the liquid. CH_4_ and CO_2_ were added to achieve a final concentration of 4% (*v*/*v*, Table 1. All incubations were performed in duplicate. Bottles were incubated in a shaking incubator at 30 °C and 135 rpm. Gas and liquid samples were taken every hour.

**Table 1:** Specification of substrate and gas additions in the batch assays to determine the potential activity of the culture

Treatment	NO_2_^−^ (mM)	NH_4_^+^ (mM)	NO_3_^−^ (mM)	CH_4_ (%)	CO_2_ (%)
NO_2_^−^ + CH_4_	0.5	–	–	4	4
NO_3_^−^ + CH_4_	–	–	0.5	4	4
NO_2_^−^	0.5	–	–	–	4
NO_3_^−^	–	–	0.5	–	4
NH_4_^+^ + NO_2_^−^	1	1	–	–	4
NH_4_^+^ + NO_2_^−^ + CH_4_	0.5	0.5	–	4	4

### Analytical methods

Liquid samples taken during activity tests were centrifuged for 1 min at maximal speed, and the supernatant was stored at −20 °C until further analysis. Ammonium was determined colorimetrically using a modified orthophataldialdehyde assay and nitrite by the Griess reaction (Ettwig et al. 2008). Nitrate was measured with the NOA280i nitric oxide analyzer (GE Analytical Instruments, Manchester, UK) after nitrite reduction to NO with acidic VCl_3_ at 95 °C. To measure the protein content of the biomass, 1.5 mL biomass samples was taken in triplicate and centrifuged for 5 min at maximal speed. The supernatant was removed, and subsequently the pellet was homogenized in 0.5 M NaOH, boiled at 90 °C for 30 min, and neutralized with 0.5 M HCl. Next, protein content was determined by the bicinchoninic acid (BCA) assay (Pierce, USA). Methane was measured using gas chromatography (HP 5890 gas chromatograph equipped with a Porapak Q column and a flame ionization detector). N_2_, O_2_, CO_2_, and N_2_O production was analyzed using gas chromatography (Agilent 6890, Porapak Q column, 80 °C) in combination with mass spectrometry (Agilent 5975c, quadrupole inert MS) (Arshad et al. 2017)

### Fluorescence in situ hybridization

Biomass samples (2 mL) from the coculture were taken on days 85, 136, and 464 and centrifuged for 5 min at maximum speed. The pellet was washed with 1 mL phosphate-buffered saline (10 mM Na_2_HPO_4_/NaH_2_PO_4_ pH 7.5 and 130 mM NaCl) and fixed using paraformaldehyde. Subsequently, FISH was performed as described before (Ettwig et al. 2008). A mixture of three probes (EUB338, EUB338II, and EUB338III, targeting most bacteria) was used to visualize the general bacterial population (Amann et al. 1990; Daims et al. 1999). A general probe targeting most archaea (ARCH0915) was used to visualize archaea (Stahl and Amann 1991). To visualize anammox bacteria, *Ca.*Methylomirabilis-like bacteria, and *Ca.*Methanoperedens-like archaea, AMX820, DBACT1043, and DBACT1043b mix and DARCH0641 probes were used, respectively (Schmid et al. 2001, Raghoebarsing et al. 2006, Ettwig et al. 2009, Schubert et al. 2011). Probes were Cy3, Cy5, or FLUOS labeled. All samples were counterstained with the DNA stain 4′,6-diamidino-2-phenylindole (DAPI). Slides were examined and images obtained by utilization of a Zeiss Axioplan 2 epifluorescence microscope equipped with a digital camera, in combination with the AxioVision software package (Zeiss, Germany).

## Results

### Anaerobic methane-oxidizing culture

**Fig. 1:**
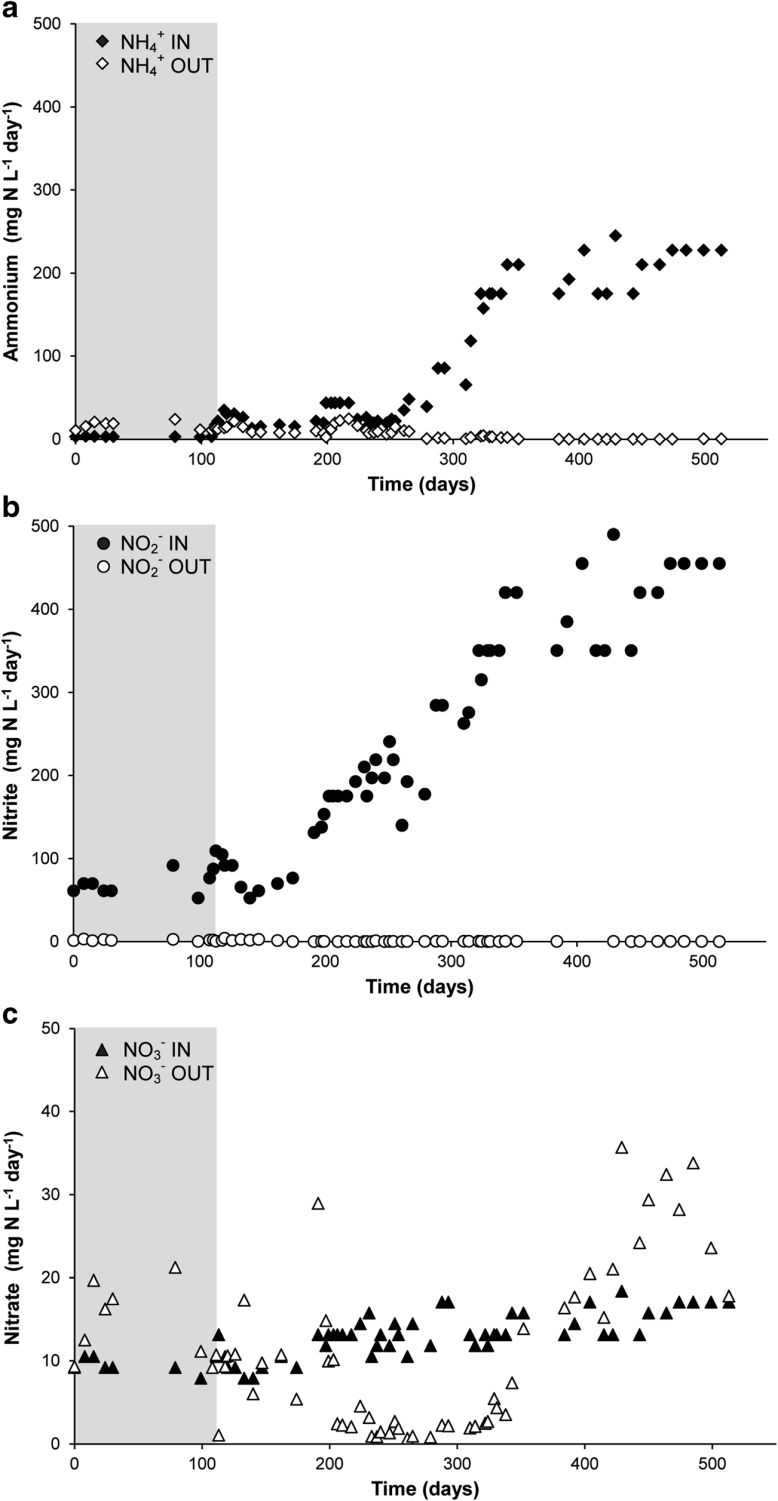
Ammonium (**a**), nitrite (**b**), and nitrate (**c**) consumption in the reactor. Closed symbols represent substrate per day fed to the reactor, open symbols show the amount of substrate per day in the effluent. In the period of *t* = 0 till *t* = 111 days (gray area), the reactor was operated as an anaerobic methane-oxidizing culture; from *t* = 111 days onwards, it was operated as an anaerobic methane- and ammonium-oxidizing coculture

**Fig. 2:**
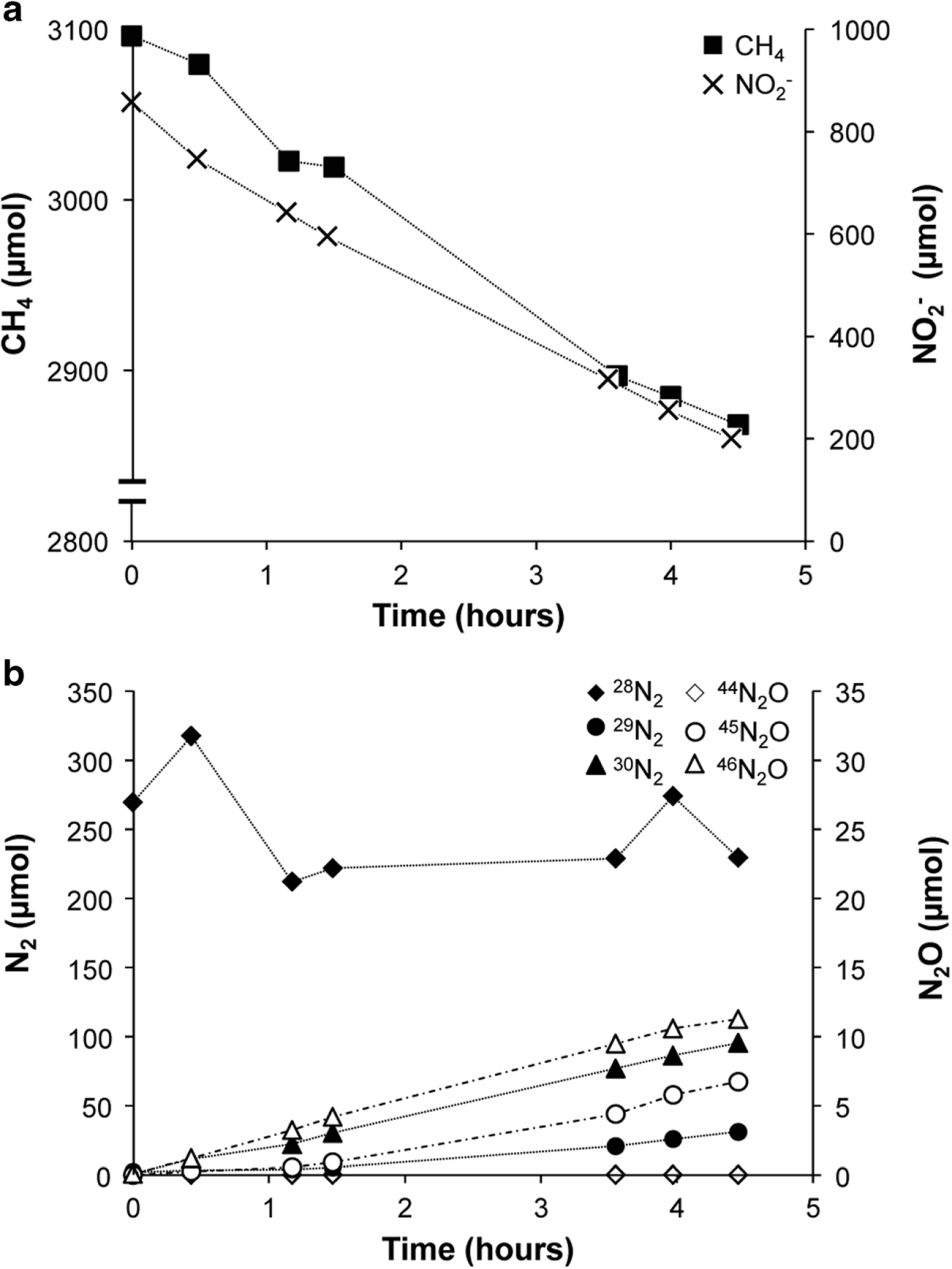
Reactor activity assay (*t* = 105 days). **a** Methane (closed squares) and nitrite (crosses) consumption. Total protein of the reactor was 9.6 ± 0.9 g. **b** Production of dinitrogen gas (closed symbols) and nitrous oxide (open symbols), ^28^N_2_/^44^N_2_O (diamonds), ^29^N_2_/^45^N_2_O (circles), and ^30^N_2_/^46^N_2_O (triangles)

A 3-L laboratory-scale SBR was started with a culture that contained both nitrate- and nitrite-dependent anaerobic methane-oxidizing(N-damo) microorganisms (Ettwig et al. 2008). In the first 110 days, the culture was supplemented with nitrate, nitrite, and methane until a stable consumption rate was achieved. Ammonium was only present as a nitrogen source. During this period, nitrite was consumed at an average rate of 63 ± 12 mg N-NO_2_^−^ L^−1^ day^−1^ (Fig. 1b), while no significant nitrate consumption was detected (Fig. 1c). On day 85, *Ca.*Methylomirabilis-like bacteria were the most abundant bacteria in the culture. Hardly any *Ca.*Methanoperedens-like archaea were visible with FISH, indicating that they had disappeared below the detection of less than a few percent of the biomass. At this phase of cultivation, also no anammox bacteria could be detected. On day 105, reactor activity was determined by supplementing the culture with methane and ^15^N-labeled nitrite. Nitrate (1.1 ± 0.04 mM) was still present in the reactor. Methane and nitrite were consumed at average rates of 53 nmol h^−1^ mg protein^−1^ and 143 nmol h^−1^ mg protein^−1^, respectively (Fig. 2a). Nitrate consumption was initially 70 nmol h^−1^ mg protein^−1^, but the consumption decreased after 1.5 h. No anaerobic ammonium consumption was observed. Consumption of ^14^N-nitrate and ^15^N-nitrite led to the production of both ^29^N_2_ and ^30^N_2_. Next to the production of dinitrogen gas, nitrous oxide production comprised about 13% of the total nitrogenous gas production (Fig. 2b).

### Anammox/N-damo coculture establishment

To establish a culture that would consume both methane and ammonium at the expense of nitrite and nitrate, 500 mL of *K. stuttgartiensis* biomass was added to the SBR on day 111 (Kartal et al. 2010). During the first 8 months after the addition, ammonium concentration in the medium could be gradually increased up to 20 mM. A stable performing culture was obtained within 363 days after the addition of the anammox cells, consuming up to 455 mg N-NO_2_^−^ L^−1^ day^−1^ and up to 228 mg N-NH_4_^+^ L^−1^ day^−1^(Fig. 1a). Nitrate consumption increased over time to 16 mg N-NO_3_^−^ L^−1^ day^−**1**^, but from around day 390 onwards, the activity of anammox bacteria resulted in net production of nitrate. As most of the ammonium was consumed via the anammox process, following the anammox stoichiometry (1:1.3:0.3, ammonium to nitrite to nitrate, Strous et al. 1997), an ammonium consumption of 228 mg N-NH_4_^+^ L^−1^ day^−1^ (*t* = 474 – 513 days) would correspond to a theoretical nitrite consumption and nitrate production by anammox bacteria of 296 mg N-NO_2_^−^ L^−1^ day^−1^ and 68 mg N-NO_3_^−^ L^−1^ day^−1^, respectively. Taking this and the nitrate in the medium feed into account, it was calculated that the total nitrate consumption by the culture was about 66 mg N-NO_3_^−^ L^−1^ day^−1^ by the end of the bioreactor operation.

**Fig. 3:**
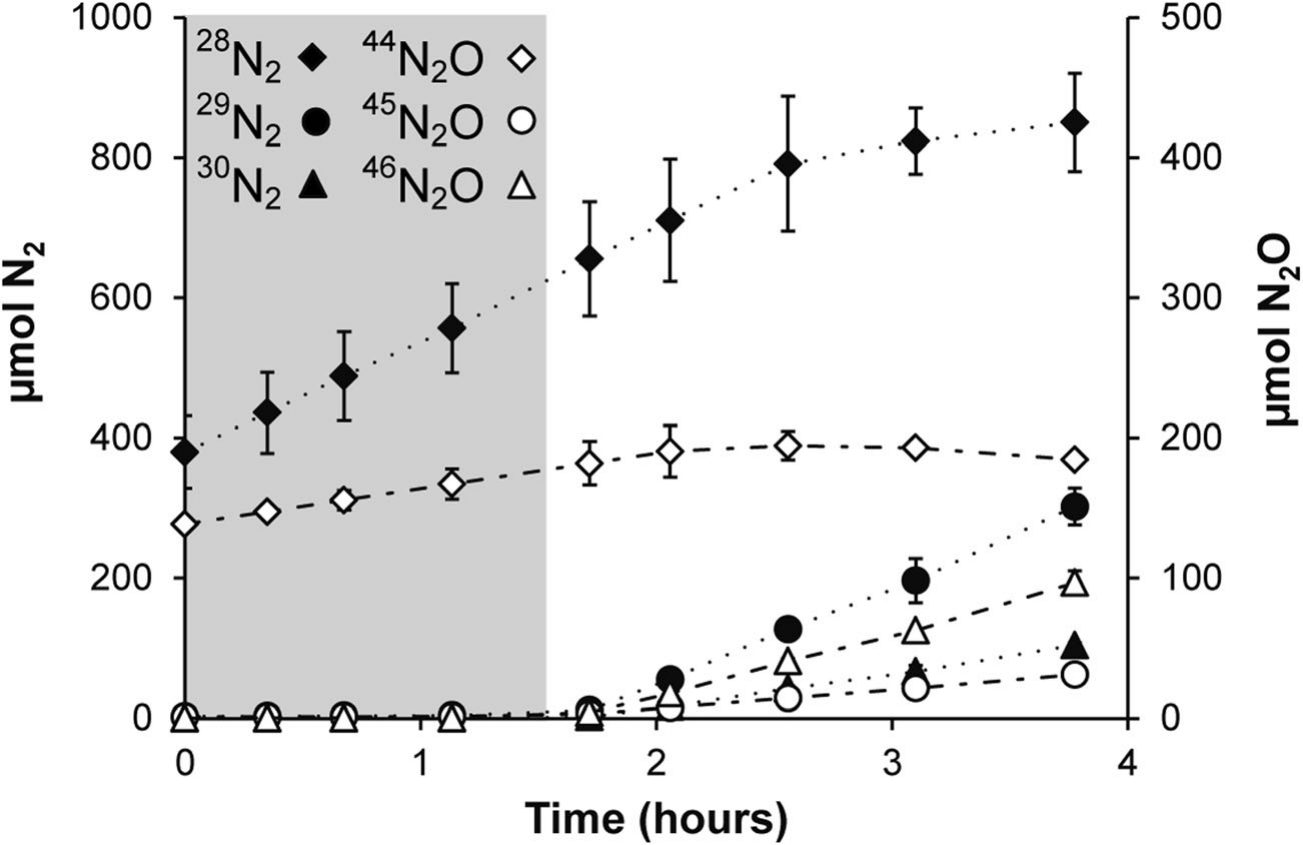
Dinitrogen gas and nitrous oxide production during activity assays (*t* = 520 days and *t* = 524 days). The closed symbols represent N_2_ and the open symbols N_2_O (diamonds: ^28^N_2_/^44^N_2_O, circles: ^29^N_2_/^45^N_2_O, triangles: ^30^N_2_/^46^N_2_O). The gray box indicates the period that medium with ^14^N-nitrite was fed to the reactor

In order to analyze the contribution of different processes to nitrogen removal within this SBR system, two reactor activity assays (on days 520 and 524) were performed in which medium supplemented with isotopically labeled ^15^N nitrite was continuously fed to the culture. The headspace was closed to measure the production of labeled nitrogen gas. Oxygen levels in the headspace remained below 30 ppm. Ammonium was consumed at a rate of 355 ± 55 nmol mg protein^−1^ h^−1^, while nitrite consumption was 664 ± 125 nmol mg protein^−1^ h^−1^. Nitrate concentration did not change throughout the experiments (results not shown). These results indicate that anammox bacteria removed about 70% of nitrite, while other processes, including N-damo, accounted for the remaining 30%. Similar results were obtained from off-gas analysis (Fig. 3), as about 75% of the produced labeled dinitrogen gas was ^29^N_2_, originating from combining labeled nitrite with unlabeled substrate (either ammonium or nitrate). Besides labeled dinitrogen gas, both ^45^N_2_O and ^46^N_2_O were produced, together accounting for 20% of the total nitrogenous gas production.

### FISH analysis

**Fig. 4:**
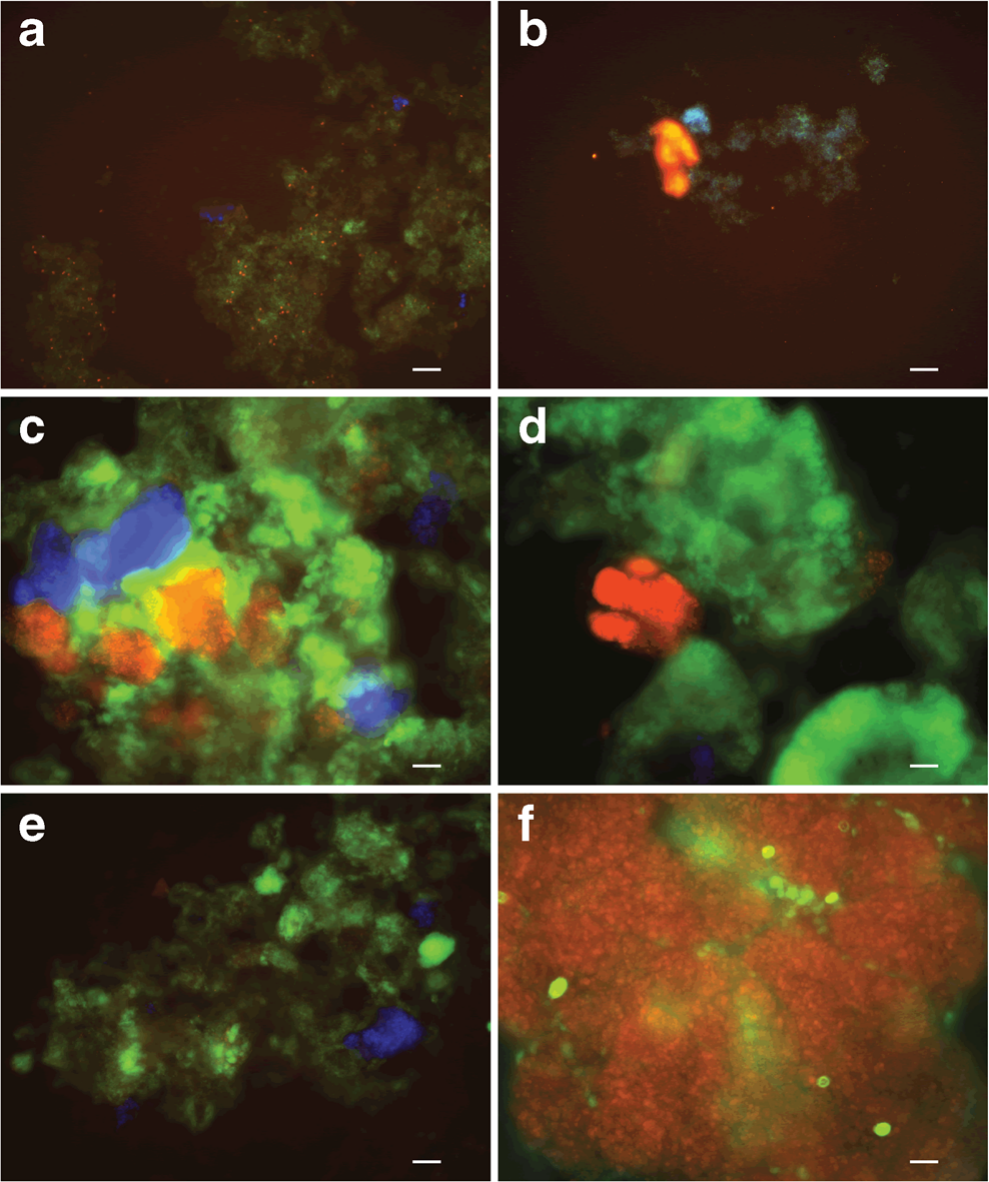
Fluorescence in situ visualization of N-damo/anammox coculture after 25 days (**a**, **b**) and 354 days (**c**–**f**) after the addition of *K. stuttgartiensis*. **a** Anammox bacteria are visible in red (Cy3, AMX820), N-damo bacteria in green (FLUOS, DAMOBACT1027), and archaea in blue (Cy5, ARCH0915). **b** 25 days after *K. stuttgartiensis* addition, most archaea were N-damo archaea (red, Cy3, DAMOARCH0641). N-damo bacteria are visible in light blue (FLUOS, DAMOBACT1027) and all bacteria in dark blue (Cy5, EUBmix). **c** 354 days after the addition of *K. stuttgartiensis*, anammox bacteria comprised 40% of the total biomass (red, Cy3, AMX820), as did N-damo bacteria (green, FLUOS, DAMOBACT1027). Archaea visible in blue (Cy5, ARCH0915) represented about 10–20% of the biomass. **d** N-damo archaea (red, Cy3, DAMOARCH0641) were the dominant archaea in the culture. N-damo bacteria are shown in green (FLUOS, DAMOBACT1027) and anammox bacteria in blue (Cy5, AMX820). **e** Of the two layers that were visible in the reactor during the settling and removal period, the bottom layer, consisting of dense granules, constitutes mainly N-damo bacteria (green, FLUOS, DAMOBACT1027) and archaea (blue, Cy5, ARCH0915). **f** In the top layer, with more floc-like biomass, anammox bacteria (red, Cy3, AMX820) were dominant. In green, some N-damo bacteria (FLUOS, DAMOBACT1027) are visible. Scale bars represent 20 μm

Community composition was studied by means of FISH on days 136 and 464, 25 and 354 days after the addition of *K. stuttgartiensis* cells, respectively (Fig. 4). Already 25 days after the addition of anammox biomass, clusters of anammox bacteria were visible in the biomass that was dominated (70–80%) by *Ca.*Methylomirabilis-like bacteria (Fig. 4a). In addition, archaeal biomass had increased up to about 5% of the biomass and mainly consisted of *Ca.*Methanoperedens-like archaea (Fig. 4b). At day 354, *Ca.*Methylomirabilis-like bacteria were no longer the dominant group and represented about 40% of the biomass. Both anammox bacteria and the *Ca.*Methanoperedens-like archaea had increased in numbers, representing 40% and 10–20% of the total biomass, respectively (Fig. 4c, d). Furthermore, upon close inspection of the biomass during the settling and removal period of the SBR, two types of biomass could be detected. The bottom layer consisted of quickly settling, dense, gray granules, which mainly consisted of *Ca.*Methylomirabilis-like bacteria and *Ca.*Methanoperedens-like archaea (Fig. 4e). In contrast, the biomass in the top layer was fluffier, less compact, and more floc-like. The flocs settled much slower but were comparable in size. Anammox bacteria comprised about 80% of this biomass (Fig. 4f).

### Potential activity batch assays

**Fig. 5:**
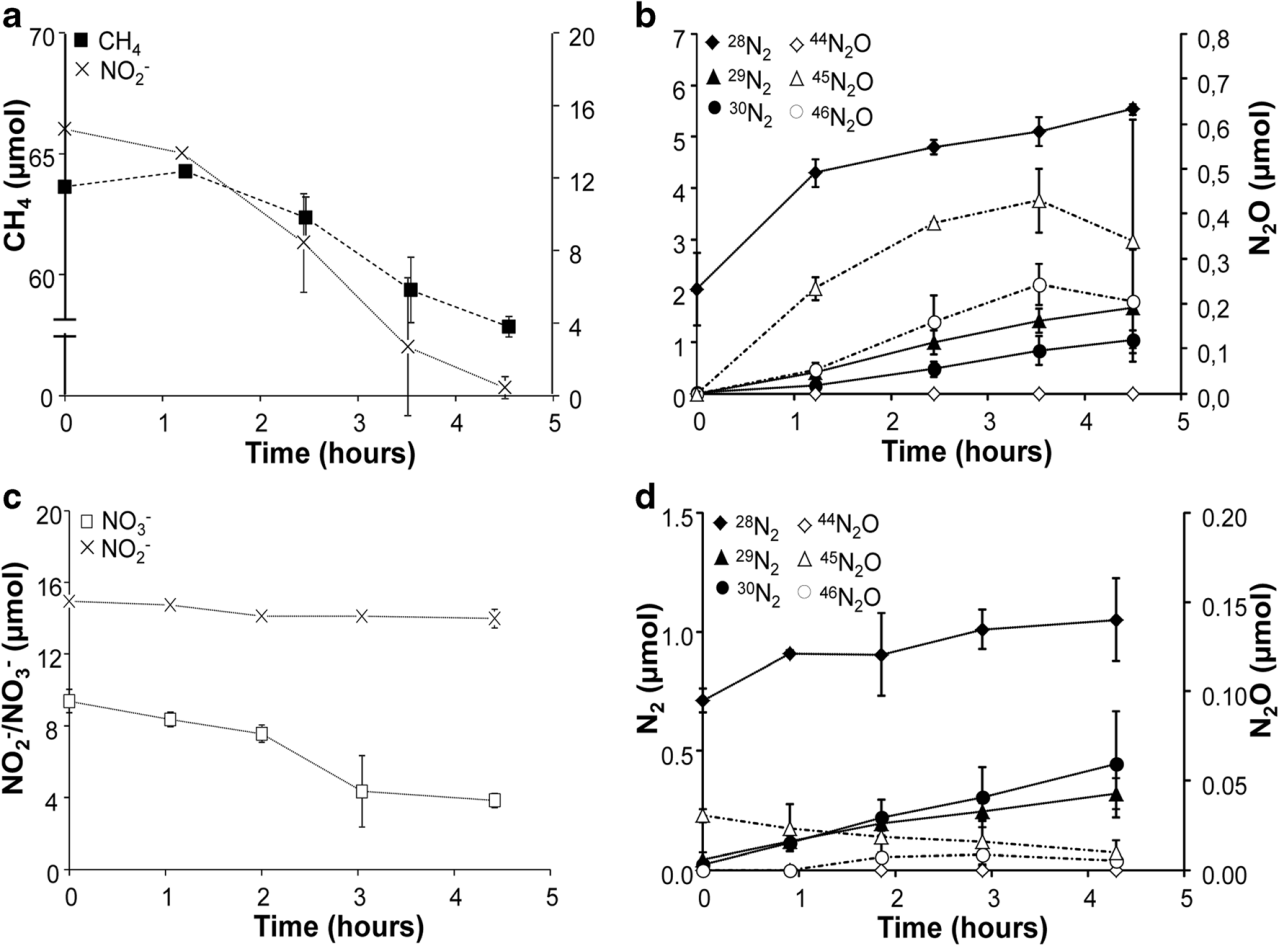
Batch incubations to determine the production of dinitrogen gas and nitrous oxide with methane and/or ^15^N-labeled nitrite as substrates. **a** Methane (closed squares) and nitrite (crosses) consumption **b** Dinitrogen gas and nitrous oxide production in incubations with methane (4%) and nitrite (0.5 mM ^15^NO_2_^−^). The closed symbols represent N_2_ and the open symbols N_2_O (diamonds: ^28^N_2_/^44^N_2_O, circles: ^29^N_2_/^45^N_2_O, triangles: ^30^N_2_/^46^N_2_O). **c** Nitrate (open squares) and nitrite (crosses) consumption in batch incubations. **d** Dinitrogen gas and nitrous oxide production in incubations with ^15^NO_2_^−^ as a substrate. The closed symbols represent N_2_ and the open symbols N_2_O (diamonds: ^28^N_2_/^44^N_2_O, circles: ^29^N_2_/^45^N_2_O, triangles: ^30^N_2_/^46^N_2_O)

**Fig. 6:**
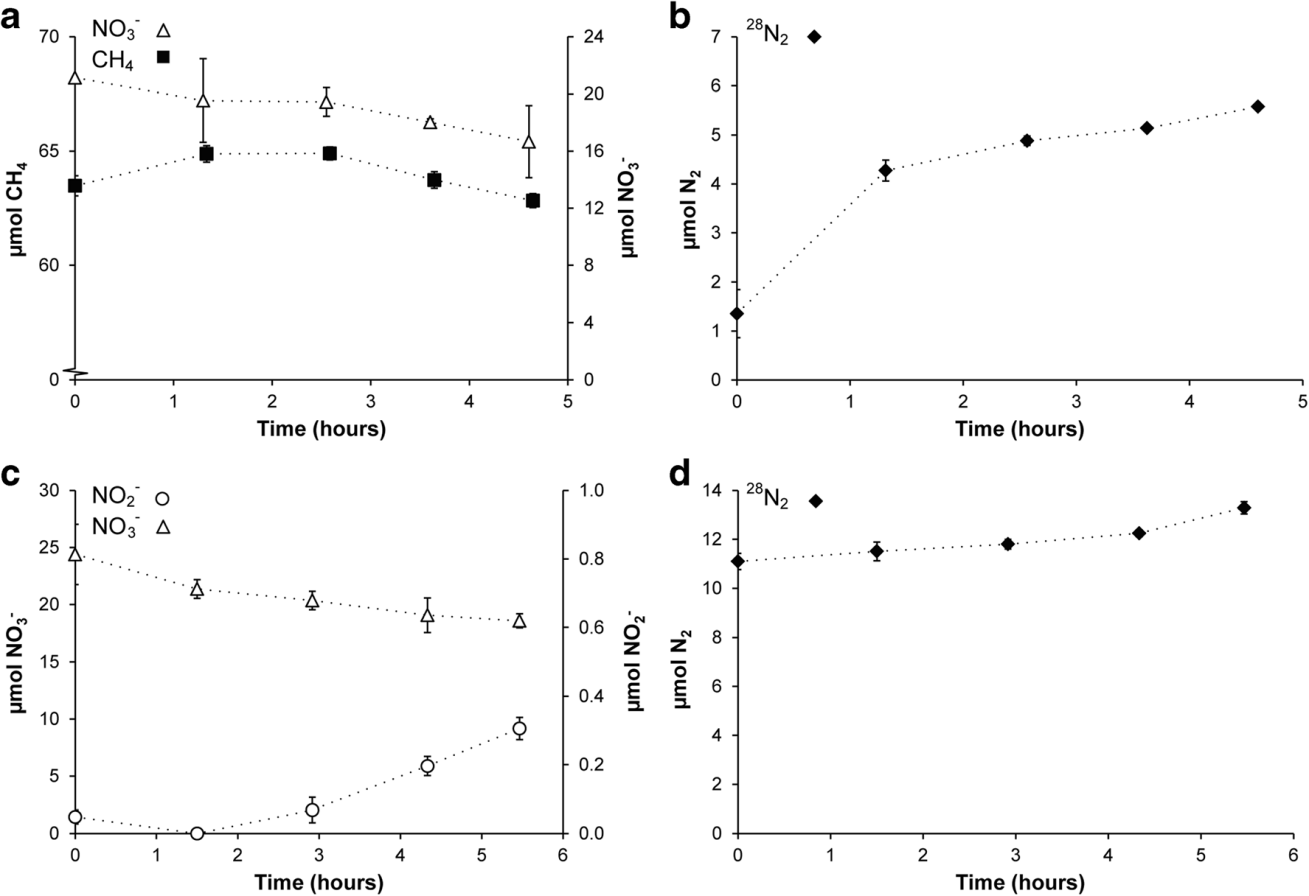
Production of nitrogenous gasses during batch incubations with methane and/or ^15^N-labeled nitrate as substrates. **a** Methane (closed squares) and nitrate (open triangles) consumption in incubations. **b** Only dinitrogen gas was formed in incubations with methane and nitrate. **c** Nitrate consumption (open triangles) and nitrite (open circles) production in incubations with nitrate (0.7 mM). **d** Only production of dinitrogen gas was detected in incubations with only nitrate

**Fig. 7:**
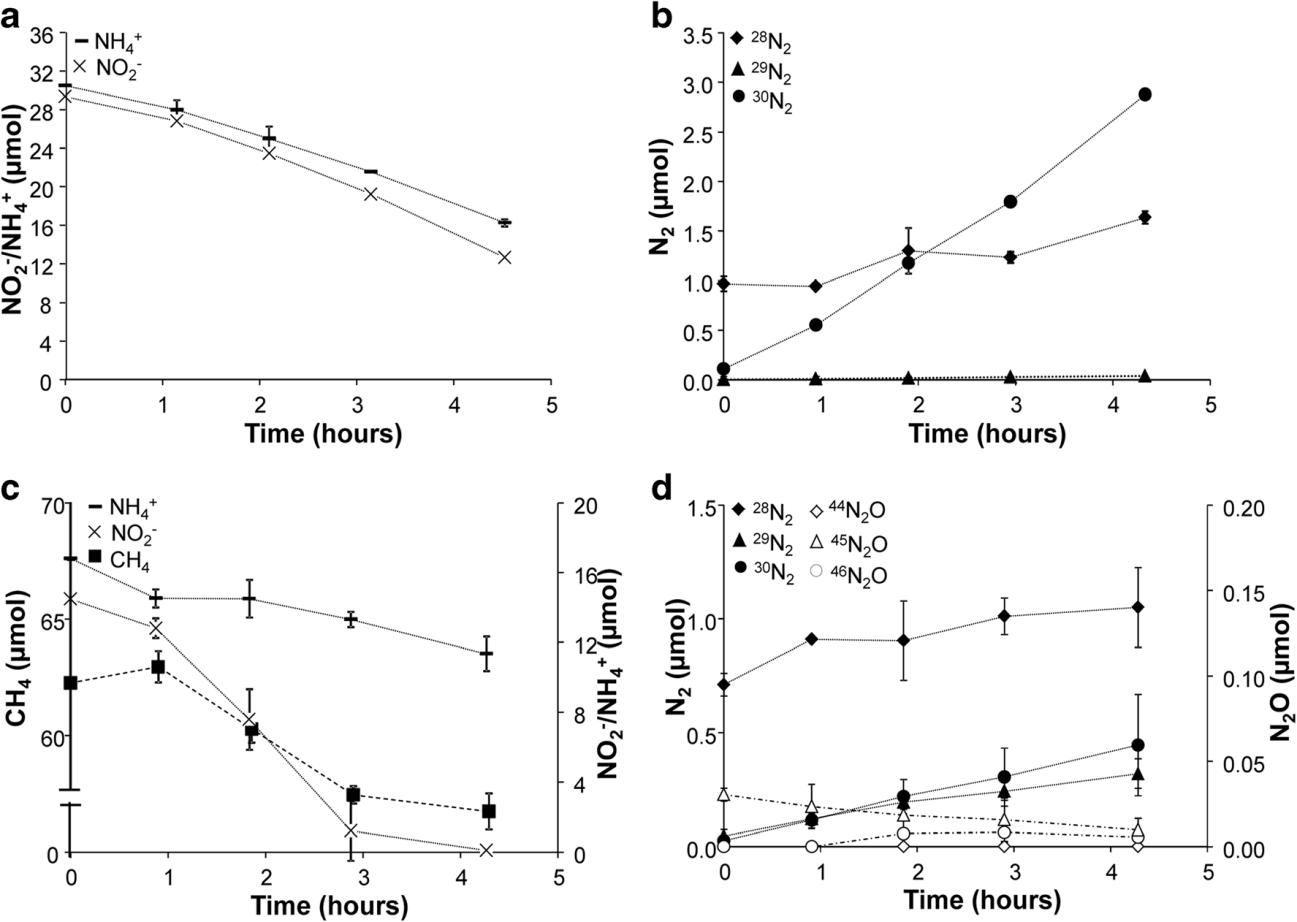
Consumption of substrates and production of dinitrogen gas and nitrous oxide during batch assays to determine the nitrite, nitrate, ammonium, and methane removal potential of the culture. **a** Ammonium and nitrite consumption in incubations with ammonium (1 mM ^15^NH_4_^+^) and nitrite (1 mM). **b** Production of ^28^N_2_ and ^29^N_2_ in incubations with ammonium (1 mM ^15^NH_4_^+^) and nitrite (1 mM). No ^30^N_2_ was formed. **c** Consumption of ammonium, nitrite, and methane in incubations with methane (4%), ammonium (0.5 mM), and nitrite (0.5 mM ^15^NO_2_^−^). Total protein was 8.3 ± 2.7 mg. **d** Dinitrogen gas and nitrous oxide production in incubations with methane (4%), ammonium (0.5 mM), and nitrite (0.5 mM ^15^NO_2_^−^)

**Fig. 8:**
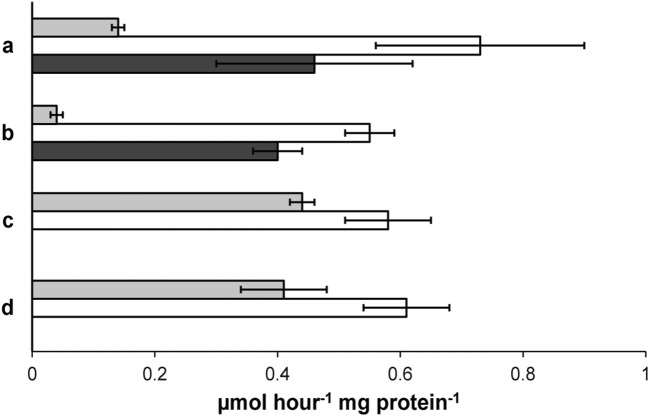
Specific consumption rates of ammonium (light gray bars), nitrite (white bars), and methane (dark gray bars) during batch assays to determine the nitrite, nitrate, ammonium, and methane removal potential of the culture. Conditions: (a) 4% methane, 0.5 mM ammonium, and 0.5 mM nitrite; (b) 4% methane, 0.25 mM ammonium, and 0.5 mM nitrite; (c) 1 mM ammonium and 1 mM nitrite; (d) 0.5 mM ammonium and 0.5 mM nitrite

The potential activity of the culture to remove nitrite, nitrate, ammonium, and methane was tested by batch assays approximately 1 year after *K. stuttgartiensis* addition. Incubations supplemented with nitrite (^15^NO_2_^−^) and methane showed high consumption rates (Fig. 5a), with maximum rates of 532 ± 179 nmol NO_2_^−^ mg protein^−1^ h^−1^ and 289 ± 62 nmol CH_4_ mg protein^−1^ h^−1^, respectively. The ratio of consumed nitrite to methane (8:4.3) was close to, but not identical to, the expected stoichiometry of nitrite-dependent methane oxidation (8:3, nitrite to methane). No ammonium was detected and background nitrate remained stable over the course of the experiment. However, ^29^N_2_, ^30^N_2_, and nitrous oxide was produced (Fig. 5b), suggesting that part of the nitrite and nitrate was consumed by N_2_O-producing microorganisms. Control incubations with only nitrite (^15^NO_2_^−^) and nitrate indeed gave a background nitrite and nitrate reduction rate of 35 ± 14 nmol NO_2_^−^ mg protein^−1^ h^−1^ and 198 ± 24 nmol NO_3_^−^ mg protein^−1^ h^−1^, respectively. Although nitrite consumption in these control incubations was low, production of labeled ^29^N_2_ and ^30^N_2_ as well as ^45^N_2_O and ^46^N_2_O could be observed (Fig. 5c, d). Incubations containing methane and nitrate as substrates showed that nitrate was also consumed in combination with methane (Fig. 6a), with average rates of 97 ± 27 nmol NO_3_^−^ mg protein^−1^ h^−1^ and 70 ± 5.8 nmol CH_4_ mg protein^−1^ h^−1^. Methane consumption exceeded the nitrate consumption based on the described stoichiometry for *Ca.* Methanoperedens archaea (methane to nitrate ratio, 2.9:4 as opposed to 1:4), suggesting that the nitrite produced in this process was most likely immediately consumed by *Ca.* Methylomirabilis bacteria. Incubations with nitrate as the sole electron acceptor only produced dinitrogen gas, and no nitrous oxide production was detected (Fig. 6b). Incubations with only nitrate did show accumulation of nitrite, but not of ammonium (Fig. 6c). Like in the incubations containing both methane and nitrate, only dinitrogen gas was produced (Fig. 6d). When ammonium (^15^NH_4_^+^) and nitrite were available as substrates, these were consumed at rates of 438 ± 23 nmol NH_4_^+^ mg protein^−1^ h^−1^ and 583 ± 65 nmol NO_2_^−^ mg protein^−1^ h^−1^ (Fig. 7a), respectively, nicely fitting the anammox stoichiometry of 1:1.3 (ammonium to nitrite). The fact that mainly ^29^N_2_ was produced (Fig. 7b) also indicated that the substrate consumption in these incubations was most likely catalyzed by the anammox process. Remarkably, the addition of methane, ammonium, and nitrite (^15^NO_2_^−^) as substrates resulted in much lower ammonium consumption rates (140 ± 11 nmol NH_4_^+^ mg protein^−1^ h^−1^) compared with incubations where ammonium was the only electron donor (Fig. 7c). In contrast, nitrite and methane consumption rates were similar to those in incubations without ammonium, namely 726 ± 166 nmol NO_2_^−^ mg protein^−1^ h^−1^ and 237 ± 80 nmol CH_4_ mg protein^−1^ h^−1^, respectively (Fig. 7c). In addition, also in these incubations, both ^29^N_2_ and ^30^N_2_ and ^45^N_2_O and ^46^N_2_O were produced (Fig. 7d). Apparently, in the presence of methane and excess nitrite, anaerobic ammonium oxidation is somewhat inhibited. This effect was even stronger at lower ammonium concentrations, while using lower ammonium concentrations in the absence of methane did not affect the consumption rate (Fig. 8). Methane itself, when added to a final concentration of 4%, does not inhibit anammox activity.

## Discussion

**Fig. 9:**
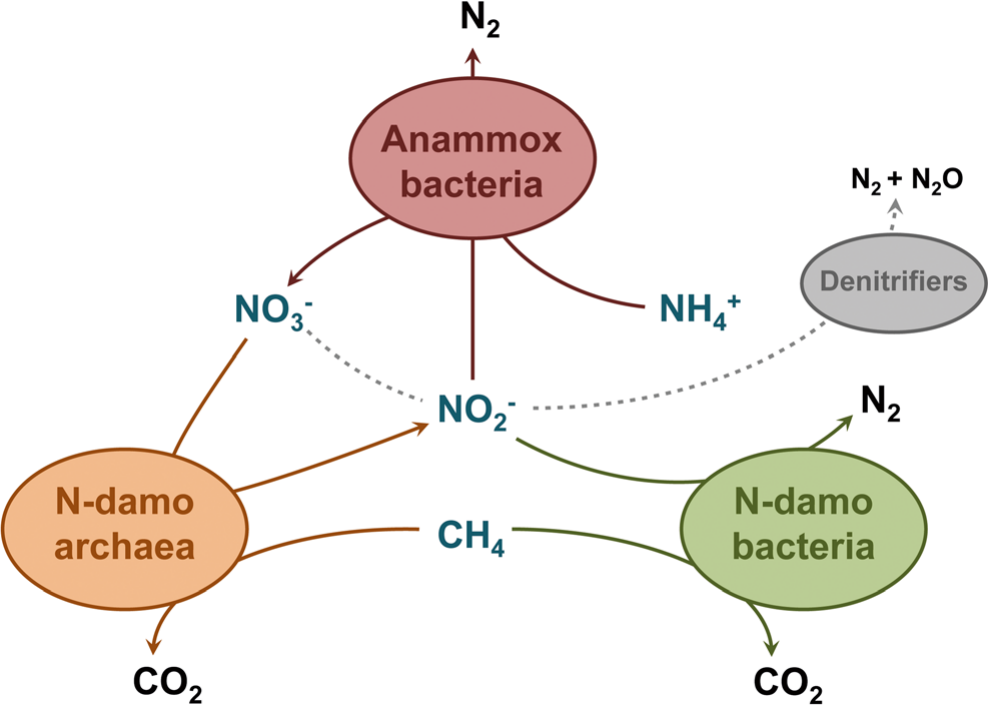
Main interactions within the SBR reactor system removing methane, ammonium, nitrite, and nitrate

By complementing an enrichment culture containing both N-damo bacteria and archaea with *K. stuttgartiensis* single cells, a stable coculture of anammox bacteria, *Ca.*Methylomirabilis-like bacteria, and *Ca.*Methanoperedens-like archaea was established. Together, these microorganisms removed methane, ammonium, nitrite, and nitrate from the supplied medium via a network of interactions (Fig. 9). Interestingly, prior to the addition of anammox cells, the N-damo enrichment culture showed somewhat lower specific anaerobic methane-oxidizing rates than N-damo enrichments reported before (Ettwig et al. 2009; Luesken et al. 2011a). After the addition of 500 mL *K. stuttgartiensis* cells, nitrite and ammonium consumption of the culture rapidly increased up to 455 mg N L^−1^ day^−1^ and 228 mg N L^−1^ day^−1^, respectively. Once a stable population was obtained, nearly all nitrate produced by anammox bacteria was consumed by *Ca.*Methanoperedens-like archaea at a rate of 57 mg N L^−1^ day^−1^ and the total nitrogen removal efficiency of the culture was as high as 97.5%. The rates for ammonium and nitrite removal reported here are amongst the higher rates for anammox/N-damo cultures (Allegue et al. 2018; Liu et al. 2019; Luesken et al. 2011a, 2011b; Shi et al. 2013; Xie et al. 2017a, b). Yet, as in these previous studies, it was shown that in a mixed anammox/N-damo culture, the presence of *Ca.*Methylomirabilis-like bacteria in large numbers does not necessarily correspond to similar contributions to nitrite consumption (Luesken et al. 2011a; Guerrero-Cruz et al. 2019). Here, *Ca.*Methylomirabilis-like and anammox bacteria both constituted about 30–40% of the culture, yet about 70% of the nitrite was removed via the anammox process similar to earlier reports (Luesken et al. 2011a). In a recent study mimicking estuarine conditions with methane, sulfide, and ammonium as electron donors for nitrate conversion, a similar biomass composition and contribution of anammox bacteria, *Ca.* Methylomirabilis, and *Ca.* Methanoperedens were observed (Arshad et al. 2017).

In full-scale wastewater treatment, influent concentrations of substrates and temperatures are varying considerably over time. In anaerobic wastewater treatment systems, methane will be present as dissolved gas in the bulk liquid. To mimic this, we used continuous gas supply resulting in saturated methane concentration in the reactor liquid. As a proof of principle, we show that an active nitrogen/methane-removing community can be established, but we realize that for applications the supply will be through the water. Previous studies used methane supply via a membrane system (Allegue et al. 2018; Chen et al. 2015; Wu and Zhang 2017). These systems may be good alternatives if biofilm growth on the membranes is carefully controlled.

In addition, ex situ batch incubations with ammonium and nitrite as substrates showed similar specific anammox activity as the activity observed in situ in the reactor. This suggests that the activity of anammox bacteria in the reactor was close to their maximum potential activity. In contrast, the specific nitrite consumption rate of *Ca.*Methylomirabilis-like bacteria in the reactor was about two times lower than the specific rates measured in the batch incubations with nitrite and methane. The batch test activities performed in this study support the previous in situ observations that these bacteria loose the competition for nitrite from anammox bacteria under nitrite limitation (Luesken et al. 2011a; Winkler et al. 2015). For future work, it may be beneficial to perform both in situ and ex situ measurements on these systems in order to better understand the microbial interactions. In addition, community analysis with FISH showed two different types of biomass layers that were visible upon settling of the biomass, indicating a physical separation of anammox bacteria and *Ca.*Methylomirabilis-like bacteria. Separation of microbiological guilds in different aggregates has been seen before (Nielsen et al. 2005). They proposed that the separation of aerobic ammonium oxidizers and anammox bacteria within a partial-nitrification anammox bioreactor into respectively smaller and bigger granules is an effect of the oxygen sensitivity of anammox bacteria. In the current system, oxygen is most likely not the cause of the separation into flocs and granules. Possibly, the formation of dense granules by N-damo bacteria and archaea prevents biomass washout (Luesken et al. 2011a, 2011b; He et al. 2015).

During batch incubations, nitrous oxide production was observed. Nitrous oxide production within N-damo cultures has been described before, and it was speculated that it originated from the denitrifying community members (Etttwig et al. 2010). In this study, only batch incubations fed with nitrite showed N_2_O production, while no N_2_O production was detected in incubations with only nitrate as an electron acceptor. In other studies, similar effects on N_2_O production by cultures after adding pulses of nitrite have been observed (Allegue et al. 2018; Itokawa et al. 2001). This might explain the observations in batch incubations, where the microbial community is subjected to pulse feeding of substrates. In addition, since the exact functions of the putative quinol-dependent NO reductases found in *Ca.* Methylomirabilis bacteria are still unknown, it cannot be excluded that these bacteria produce N_2_O under certain conditions (Etttwig et al. 2010; Wu et al. 2011; Zhu et al. 2017; Graf et al. 2018; Versantvoort et al. 2018).

As observed in previous studies and here, competition for nitrite is an important regulator of anammox/N-damo systems, but also other chemical compounds, such as trace elements, could play a role in this. Recently, it has been reported that the concentration of available iron and copper might play a role in the competition between anammox and *Ca.* Methylomirabilis bacteria (He et al. 2015; Lu et al. 2018). Concentrations of iron and copper used in the current study were 10 μM and 2.5 μM, respectively. More research is needed to determine how the use of medium containing more iron and copper could influence the community and the competition for substrates in an anammox/N-damo coculture. Remarkably, also the presence or absence of rare earth elements may determine which *Ca.* Methylomirabilis bacteria will become dominant in the culture. Recently it was shown that inclusion of cerium in the trace elements of a *Ca.* Methylomirabilis enrichment culture resulted in the appearance of a new species named *Ca.* Methylomirabilis lanthanidiphila that only had a xoxF-type methanol dehydrogenase encoded in its genome (Versantvoort et al. 2018).

In addition, it remains unclear why anammox bacteria seemed not active in batch incubations with ammonium, nitrite, and 4% methane, while winning the competition for nitrite under methane saturated conditions in the reactor. Batch incubations with the *K. stuttgartiensis* biomass in the presence of 4% methane did not show any inhibition of anammox activity, indicating that N-damo microorganisms might influence anammox activity when nitrite is not limiting. Also, to apply an anammox/N-damo system in anaerobic wastewater treatment plants, nitrite will have to be produced from ammonium via partial nitrification, as is the case in partial nitritation-anammox systems (van Kessel et al. 2018). Next to ammonium oxidizers, other aerobic microorganisms like aerobic methanotrophs and heterotrophs might start to grow, while N-damo and anammox might be inhibited (Castro-Barros et al. 2017; Guerrero-Cruz et al. 2018; Luesken et al. 2012; Wu and Zhang 2017). Several studies have explored the feasibility of such a combined nitritation-anammox-N-damo system by modeling (Bhattacharjee et al. 2016; Castro-Barros et al. 2017; Chen et al. 2015; Winkler et al. 2015). Now the next step would be to test the potential of such a system in laboratory-scale reactors.
